# A Guide to Different Intensities of Exercise, Vaccination, and Sports Nutrition in the Course of Preparing Elite Athletes for the Management of Upper Respiratory Infections during the COVID-19 Pandemic: A Narrative Review

**DOI:** 10.3390/ijerph19031888

**Published:** 2022-02-08

**Authors:** Hamid Agha-Alinejad, Amir Hossein Ahmadi Hekmatikar, Ruheea Taskin Ruhee, Mahdieh Molanouri Shamsi, Masoud Rahmati, Kayvan Khoramipour, Katsuhiko Suzuki

**Affiliations:** 1 Department of Physical Education and Sport Sciences, Faculty of Humanities, Tarbiat Modares University, Teheran 1411713116, Iran; halinejad@modares.ac.ir (H.A.-A.); a.ahmadihekmatik@modares.ac.ir (A.H.A.H.); molanouri@modares.ac.ir (M.M.S.); 2 Future Innovation Institute, Waseda University, Shinjuku 162-0041, Japan; ruhee@fuji.waseda.jp; 3 Department of Physical Education and Sport Sciences, Faculty of Literature and Human Sciences, Lorestan University, Khoramabad 6816785468, Iran; rahmati.mas@lu.ac.ir; 4 Institute of Neuropharmacology, Neuroscience Research Center, Department of Physiology and Pharmacology, Afzalipour School of Medicine, Kerman University of Medical Sciences, Kerman 7616914115, Iran; 5 Student Research Committee, Kerman University of Medical Sciences, Kerman 7619813159, Iran; 6 Faculty of Sport Sciences, Waseda University, Tokorozawa 359-1192, Japan

**Keywords:** athlete’s immune system, training intensity, COVID-19, performance

## Abstract

Elite athletes use high-intensity training to maintain their fitness level. However, intense training can harm the immune system, making athletes suspectable to COVID-19 and negatively affecting their performance. In addition, the diet of athletes should be appreciated more as it is another influencer of the immune system, especially during the COVID 19 pandemic. The other important issue elite athletes face currently is vaccination and its possible intervention with their training. The present study attempts to discuss the impact of different training intensities, nutritional strategies, and vaccination on the immune system function in elite athletes. To this end, Scopus, ISC, PubMed, Web of Science, and Google Scholar databases were searched from 1988 to 2021 using the related keywords. The results of our review showed that although high-intensity exercise can suppress the immune system, elite athletes should not stop training in the time of infection but use low- and moderate-intensity training. Moderate-intensity exercise can improve immune function and maintain physical fitness. In addition, it is also better for athletes not to undertake high-intensity training at the time of vaccination, but instead perform moderate to low-intensity training. Furthermore, nutritional strategies can be employed to improve immune function during high-intensity training periods.

## 1. Introduction

**Figure 1:**
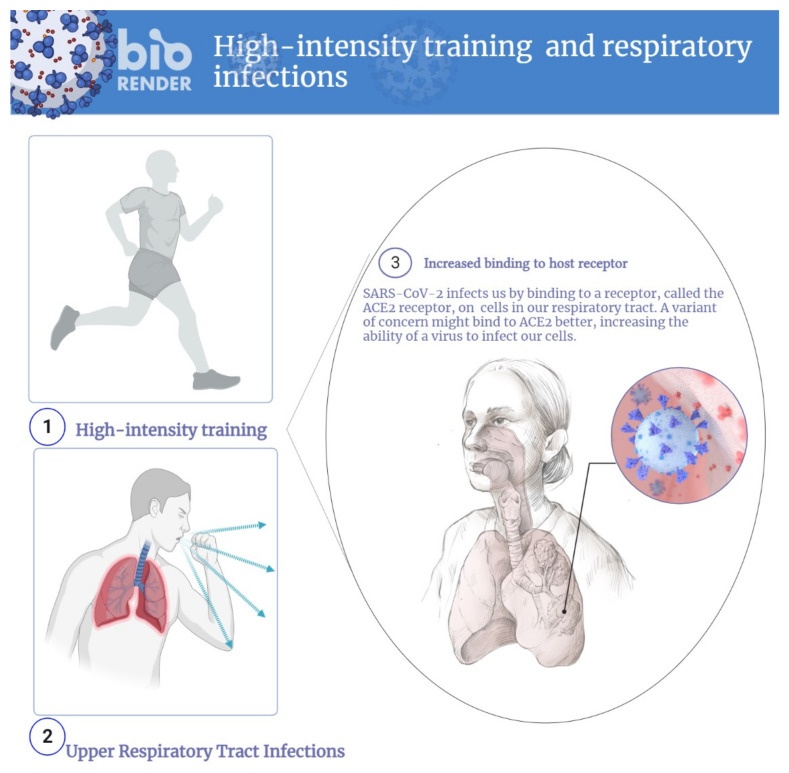
High-intensity exercise and upper respiratory tract infections.

The immune system plays a significant role in protecting the human body from bacterial and viral infections and other environmental contaminants [1]. Several factors affect the function of the immune system, including age, gender, nutritional habits, medical status, exercise training, diet, and fitness level [2,3]. Both in its acute and chronic forms, physical exercise performance significantly alters the immune system function [4,5]. Current evidence suggests that regular exercise can exert both positive and negative effects on the normal functioning of the immune system [4,5]. It has been well documented that the modulation of the immune response by exercise depends on several factors, including regularity, intensity, duration, and type of exercise [6]. Accordingly, excessive, prolonged, high-intensity exercise may impair immune system function [7]. Notably, compared with non-elite athletes, higher training intensities are frequently employed by elite athletes to improve their physical fitness—as this training is needed for winning national and international medals—which may make them sensitive to infections [8]. Several studies indicate that upper respiratory tract infections (URTI) are common among elite athletes performing intense exercise [9,10]. URTI is an acute infection in the upper respiratory tract, including the nose, sinuses, and pharynx [11]. Athletes who perform strenuous exercise training are 2–6 times more likely to develop URTI if exposed to pathogens [12]. The risk of URTI increases among marathon runners between 1 and 2 weeks after the event due to reduced neutrophil function (Figure 1) [13]. Furthermore, a URTI association has been reported with COVID-19 [14]. These findings highlight the important issue that intense training and competition in elite athletes can suppress their immune system, predisposing them to COVID-19.

The immune system plays a vital role in the success of elite athletes [15]. Immune depression in elite athletes taking part in strenuous sports can harm their performance [15]. In addition, due to the COVID-19 outbreak starting in 2020, the importance of the immune function should be particularly appreciated in athletes. Although sudden cessation of exercise can lead to a marked decline in immune function [8], in the case of impaired immunity and respiratory infections, exercise should not be stopped abruptly in elite athletes.

After undergoing home quarantine, many athletes have resumed intense training to maintain their physical fitness levels for upcoming competitions. However, studies have shown that after exercise cessation due to the COVID-19 pandemic, retraining should be started with caution [16]. Another big challenge that elite athletes face is resuming high-intensity training after being affected by the coronavirus. It has been shown that a high level of stress and anxiety during the infection may lead to athletes making wrong decisions about the best training intensity after returning to the athletic field [17]. As the sudden onset of intense exercise and training is associated with immunodepression [18], returning to sports activities and competition events should be supervised closely by their coaches and teams.

Nutrition is another critical factor influencing immunity because macro- and micro-nutrients are involved in multiple immune processes [19]. Nutritional deficiencies can compromise the immune system response and increase susceptibility to infections, including COVID-19, especially in elite athletes [20]. Therefore, elite athletes who want to perform high-intensity exercises during the COVID-19 pandemic should follow proper nutritional strategies. Additionally, elite athletes should receive vaccines to improve their immune system, thus helping them fight against COVID-19. One concern about elite athlete vaccination is continuing high-intensity training when they receive vaccine shots [21]. In the present study, an attempt is made to discuss the impact of different training intensities, nutritional strategies, and vaccination on the immune system function in elite athletes.

## 2. Analysis Method

### 2.1. Search Strategy

A literature search was conducted on Scopus, Web of Science, ISC, Pub-Med, and Google Scholar databases from 1988 to 2021 to find the related articles using several keywords, i.e., low-intensity exercise and immune system, moderate-intensity exercise and immune system, high-intensity exercise and immune system, low-intensity exercise and athlete recovery, moderate-intensity exercise and athlete recovery, high-intensity exercise and athlete recover, sports nutrition and athlete immune system, athlete stress and immune system, athlete sleep and immune system, carbohydrate and athlete immune system, vaccination and elite athletes.

### 2.2. Inclusion and Exclusion Criteria

In this review, we included any randomized single or double-blinded case-control, cohort, or experimental study with an intervention involving or an objective to explore the relationship between different intensities of exercise and immunity in athletes. Studies with unclear statements or results, not mentioning exercise intensity, and do not have a control group, were excluded. In addition, studies published in languages other than English were excluded.

## 3. Results and Discussion

### 3.1. Low-Intensity Exercise and Athletes’ Immune Systems

There is very little research on low-intensity training and athletes’ immune systems. Low-intensity training (e.g., below the first ventilatory threshold, at stable lactate concentrations < 2 mM or with an intensity of less than 37–45% VO_2max_), is also referred to as long slow distance training or zone-1 training. Steensberg et al. [22] reported increased IL-6 and IL-10 after 3 h and 26 min of low-intensity exercise. Mee-Inta et al. [23] concluded in their review study that low-intensity exercise can reduce inflammation. It has also been reported that low-intensity exercise (less than 60% VO_2max_) for less than 60 min can reduce inflammation and improve immune function [24]. Tenorio et al. [25] examined the effect of low- versus high-intensity exercise training on inflammation and endothelial dysfunction biomarkers in adolescents with obesity in a 6-month randomized exercise intervention study. Interestingly, they found that high and low exercise intensities can improve immune function (neutrophils, monocytes, tumor necrosis factor-alpha).

Generally, low-intensity exercise has been considered to be a good strategy for elite athletes’ recovery and reducing post-competition stress [26]. Taken together, these studies indicate that low-intensity exercise can create a positive adaptation in the immune system (Table 1).

### 3.2. Moderate-Intensity Exercise and Athletes’ Immune Systems

Moderate-intensity exercise performed between first and second lactate or ventilatory threshold (i.e., zone-2) and causes accumulated lactate levels [28]. Performing exercise with an intensity of 45 to 65% VO_2max_ is considered moderate-intensity exercise.

MacIntosh et al. [29] reported that regular moderate-intensity training can reduce inflammation, and increase IL-10 and T-cell function. Other studies showed that if moderate-intensity exercise is performed for more than 60 min, it can increase inflammation [30]; otherwise it decreases inflammation [26]. It has been reported that IL-10, an anti-inflammatory cytokine, increases in both intense and moderate-intensity exercise [31]. However, moderate-intensity exercise has been shown to reduce cytokine storms and increase white blood cells, lymphocytes, and T cells [32].

In another study, Fashi et al. [33] concluded that four weeks of aerobic exercise reduced inflammation in the lung. Shiri et al. [34] examined the effect of six weeks of endurance training on tumor tissue IL-10 levels in breast cancer-bearing mice, and reported a significant increase in IL-10 levels. 

Taken together, these studies demonstrate that moderate-intensity exercise is the best strategy in preventing suppression of the immune system (Table 2).

### 3.3. High-Intensity Exercise and Athletes’ Immune Systems

**Figure 2:**
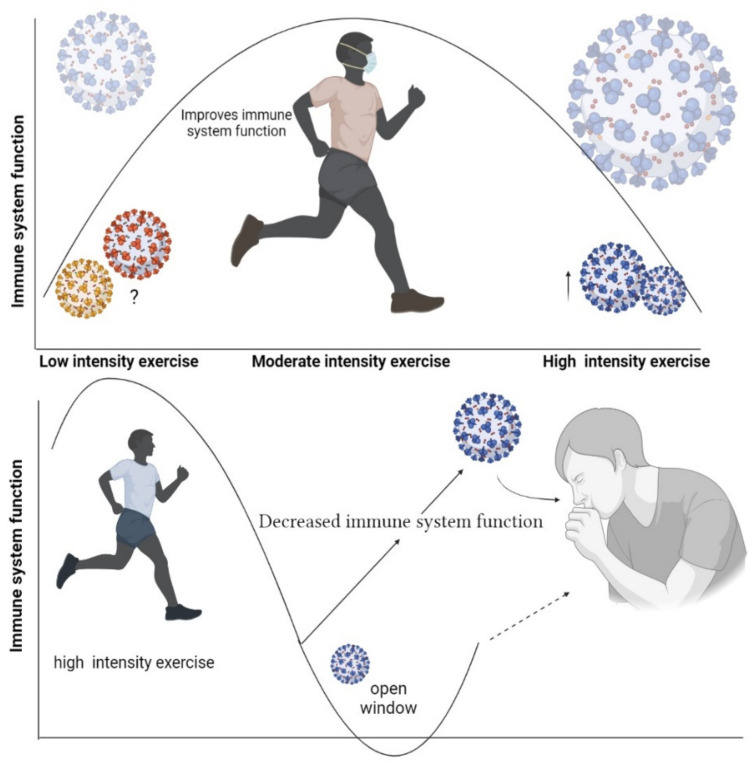
Open window theory after high-intensity training.

High-intensity exercise refers to an intensity higher than 70% of VO_2_max [35]. High-intensity or “zone-3” training (e.g., >4 mmol lactate/L blood, >90% maximal heart rate) involves intermittent intervals exercises (short, high-intensity sprints) [36]. Some markers of the immune function change within a few days after long-term intense endurance physical exercise. Neutrophils and NK cell functions, salivary immunoglobulins A (IgA), and some types of inflammatory macrophages are shown to undergo negative changes following this kind of exercise training [37]. In addition, based on the “open window” theory, 3 to 72 h after intense exercise an infectious agent may be able to invade the host body, thus increasing the risk of opportunistic infections [38] (Figure 2).

## 4. Respiratory Infections and Exercise

Evidence suggests that chronic exercise can increase upper respiratory tract infections in athletes [55]. The risk of athletes getting respiratory infections after intense exercise training is 7 times more than inactive individuals and 2 times more than active individuals [55]. Recurrent infections in athletes can be dangerous during a coronavirus pandemic [56].

Svenndsen et al. [57] reported that intense skiing increases the risk of infection by 3 times compared to recreational skiers. Nieman & Wentz [58] reported that intense physical activity lasting less than 60 min can suppress the immune system. In line with these results, other studies have reported that intense exercise lasting more than 60 to 90 min can suppress immune function [4,8]. It has also been found that elite athletes who perform intense exercise to prepare for professional competitions may be more susceptible to infectious diseases [59]. Thus, elite athletes are more prone to URTI during preparation for a professional competition. Being overtired may make this situation worse. Mackinnon et al. [60] reported that after 4 weeks of high-intensity training in swimmers, 33% of athletes showed symptoms of restlessness and 42% self-reported symptoms of URTI. During intense exercise, the activities of the lymphatic system are disrupted, which can negatively affect the immune system [61].

Allergic rhinitis is common among athletes due to regular high-intensity exercise [62]. To prepare for competitions, elite athletes undertake high-intensity training chronically trying to improve their physical fitness. It has been reported that, of 216 Olympic athletes, 56% had a history of conjunctivitis and rhinitis [62]. Allergic rhinitis impairs physical performance in professional athletes by affecting sleep, decreasing the ability to concentrate, or reducing physical fitness [62]. Continuous exposure to allergic rhinitis can increase the number of lymphocytes, eosinophils, neutrophils, basophils, and other leukocytes. This can cause the airways to overreact and eventually lead to fibrosis [63]. Allergens can also stimulate the airway epithelium to release IL-25, IL33, and thymic stromal lymphopoietin (TSLP). These cytokines can activate innate submucosal lymphocytes (ILC2) and release IL-4, IL-5, IL-9, and IL-13 [63], thereby causing airway wall remodeling, bronchial hyperresponsiveness, and goblet cell metaplasia [63]. These data, along with the open window theory, suggest that elite athletes are at high risk for COVID-19, highlighting the importance of vaccination and health care during infection.

## 5. Management during Athletes Infection 

It has been found that viral infections can happen due to intense training in elite athletes, leading to a decrease in aerobic performance, especially among those affected by COVID-19 [64]. It has also been shown that athletes develop a fever during infection, and their muscle strength decreases [65]. The first step in managing athletes’ infections is to reduce exercise intensity and use nutritional strategies [19]. Infection management in athletes can be divided into two categories: (a) strategies for severe infections, and (b) strategies for athletes with minor symptoms. Athletes who develop viral infections due to intense exercise and have a severe physiological condition must have active rest (i.e., low-intensity exercise) [66]. Protein catabolism increases in this situation [67]; thus, low-intensity resistance training and protein supplementation are the best choice [68]. For the second group, aerobic and moderate-intensity training can be appropriate. It is recommended that athletes do not stop exercising when they develop URTI or COVID-19 as it makes the situation worse because the sudden cessation of exercise due to illness can further weaken the immune system.

## 6. How Can Athletes Get Vaccinated While They Continue Training?

In the previous sections, the importance of vaccination in elite athletes was highlighted. One concern with athlete vaccination is that the first dose may have short-term side effects. These side effects vary depending on the type of vaccine [21]. It is recommended that athletes reduce exercise intensity when receiving the first dose of the vaccine [69]. Athletes performing moderate- to low-intensity exercise training do not need to reduce exercise intensity [69]. It has been found that nearly 94% of athletes experience arm pain, general fatigue, and fever after receiving the vaccine for 2 days [70]. Therefore, it can be suggested that, during this period, the intensity of training should be reduced in athletes who undertake high-intensity training. After the symptoms disappear, the intensity can gradually increase intensity. High-intensity training can continue until a day before the second dose, when training intensity should be reduced again. Elite athletes have been shown to experience headaches, chills, fever, and muscle aches for 1 to 3 days after receiving the second dose [70,71]. Thus, the exercise intensity should kept low until the fourth day after receiving the second dose and then increase gradually [21,72].

## 7. The Importance of Nutrition

Adherence to diets and dietary supplements during high-intensity interval training can minimize the suppression of the immune system [73].

It has been shown that carbohydrate consumption may improve immune function (Table 3), further improving sports performance [74]. Carbohydrates can increase performance by increasing blood glucose, and glucose can decrease cortisol and increase IL-10 levels [75], leading to improved immune response [75]. It is recommended that athletes use 30–70 g of carbohydrates per hour depending on the intensity and duration of exercise [76]. Additional recommendations to help immune function in athletes include (a) avoiding sudden dietary changes, (b) receiving 50% of total daily calories from carbohydrate, and (c) consuming vitamin C and D [49]. In agreement with this, Gunde [73] stated that, for sports competitions that last more than 90 min, the recommended dose is between 30 and 60 g per hour. It is clear that, with increasing the duration of sports competitions, the amount of carbohydrate intake should increase. For instance, Jagger et al. [77] stated that between 60 and 90 g of carbohydrate per hour should be consumed for competitions lasting more than 2.5 h. 

In addition to carbohydrates as the primary fuel for athletes, protein is needed to maintain/increase their muscle mass. The amount of protein an athlete needs depends on their type of activity. The general recommendation is an average intake of 1.3 to 1.8 g per kg per day [87]. A protein-rich diet is consumed to increase muscle mass, hypertrophy, strength, and fat loss, and improve recovery and performance. However, both positive and negative results have been obtained due to chronic protein supplementation or a protein-rich diet intake. Data from a systematic review and meta-analysis showed that protein supplementation during resistance exercise training does not improve muscle strength, size, or functional ability [88].

Herbal supplements or complementary and alternative medicines are familiar due to their unique medicinal properties. They also help to enhance the immune system by adding isolated vitamins and minerals. A wide range of phytonutrients, such as polyphenols, flavonoids, carotenoids, sulforaphane, curcumin, sulfides, and plant sterols, are found in herbs [76]. Some of these phytonutrients show protective effects against exercise-induced endotoxemia, and reduce exercise-induced inflammation and oxidative stress by stimulating the activity of protective enzymes such as phase 2 detoxification enzymes and antioxidative enzymes [89,90].

## 8. Conclusions

The results of our review showed that although high-intensity exercise can suppress the immune system, elite athletes should not stop training during the period of infection, but use low- and moderate-intensity training. Moderate-intensity exercise can improve immune function and maintain physical fitness. In addition, it is also better for athletes not to perform high-intensity training at the time of vaccination but instead perform moderate- to low-intensity training. Furthermore, nutritional strategies can be employed to improve immune function during high-intensity training periods.

## Figures and Tables

**Table 1:** A review of low-intensity exercise on the immune system.

Authors	Intensity	Results	Source
Mee-Inta et al. (2019)	Low	Increases IL- 10 and IL-6 and decreases cortisol levels	[23]
Tenorio et al. (2019)	Low	Increases IL- 10 levels and T cell numbers. Improves immune system function	[25]
Petersen and Pedersen. (2005)	Low	Decreases IL- 1β and TNF-α levels. Improved immune system function	[27]
Steensberg et al. (2003)	Low	Increase in IL- 10 and T cells. Improves immune system function	[22]

**Table 2:** Effect of different intensities of exercise on immune system.

Author	Type of Exercise	Intensity of Exercise	Results	Reference
Raines et al. (2020)	Resistance	45%, 75% and 95% 1RM	Increase in IL- 6 at 75% and 95% 1RM No change in 45% 1RM	[39]
Xiao et al. (2020)	Resistance and aerobic	Walking: 5 days a week, 30 min with an average intensity of 45 VO_2_max for 12 weeks. Resistance training: 2 sets of resistance training with banding and Borg scale between 12 to 13. Intensity was higher than 60% 1RM.	High-intensity resistance training: Increases IL-1 and TNF-α and C-reactive protein.Walking: Increases IL-10	[40]
Scheffer and Latini (2020)	High-intensity	Review study	Exercise intensity 46–63% VO_2_max: Increases anti-inflammatory cytokines including IL-10, IL-6, and IL-7. 64 to 100% VO_2_max increases L-1β, IL-6, TNF-α, IL-17A and IL-15	[41]
Dixit (2020)	Aerobic	Review study	45 to 60% VO_2_max increases antipathogen activity, recirculation of immunoglobulins, anti-inflammatory cytokines, neutrophils, NK cells, cytotoxic T cells, and immature B cells.	[42]
Highton et al. (2020)	Aerobic	20 min walking at 60–70% VO_2_peak	Increase notrophil and monocyte	[43]
Sitlinger et al. (2020)	Moderate intensity	Review study	Increases T cells, natural killer cells, neutrophils, monocytes, and B cells	[44]
de Souza et al. (2018)	Aerobic	Running on a treadmill at a speed of 3.0 km/h in increments of 1.0 km/h every minute until voluntary exhaustion Running with moderate intensity for 20 min at 65–75% of HRpeak	Increases IL-6, IL-4, and interferon-γ. Decrease IL-6, IL-4, and interferon-γ.	[45]
Hajizadeh et al. (2018)	Aerobic	Over the first 12 weeks of the intervention, walked or jogged on a treadmill at 45–55% of their VO_2_max (25–30 min/day, 3–4 days/week), and after that exercised by an intensity of 56–69% of VO_2_max (40–45 min/day, 4–6 days/week) over the final 12 weeks.	Decrease IL-1β, IL-6, IL-8, TNF-α and increase IL-10	[46]
Durrer et al. (2017)	Aerobic	7 × 1 min at ~85% maximal aerobic power output, separated by 1 min of recovery on a cycle ergometer.	Increases TNF-α	[47]
Szlezak et al. (2016)	Aerobic and anaerobic	Systematic review	Exercise with an intensity of 45 to 65% VO_2_max: increases T cells, natural killer cells, neutrophils, monocytes, and B cells .Exercise with an intensity of 64 to 100% VO_2_max: increases L-1β, IL-6, TNF-α, IL-17A and IL-15	[48]
Dorneles et al. (2016)	Aerobic and anaerobic	10 × 60 s (85–90%P_Max_)/75 s (50%P_Max_) 10 × 60 s (70–75%P_Max_)/60 s (50%P_Max_)	increases IL-1ra, IL-6 and IL-8. increases IL-10	[49]
Sarir et al. (2015)	Anaerobic	Running on a treadmill for five days, 10 min/day at a 10 m/min speed. Then, six sessions per week at 95–100% VO_2_max for six weeks. Active rest was performed between intervals for 60 s at 16 m/min.	increases IL-6 and TNF-α	[50]
Neves et al. (2015)	Anaerobic and aerobic	High-intensity exercise (80% VO_2_peak), low exercise intensity (40% VO_2_peak).	High-intensity exercise: increases in leukocyte, Lymphocyte, and monocyte. Low-intensity exercise: does not produce any changes.	[51]
Zwetsloot et al. (2014)	Anaerobic	Two weeks of cycle ergometer, 3 session per week (8–12 intervals; 60-s intervals, 75-s active rest) at 100% VO_2_max.	Increases IL-6, IL-8, IL-10, monocyte and TNF-α.	[52]
Gholamnezhad et al. (2014)	Anaerobic and aerobic	Moderate training (20 m/min, 30 min/day, 6 days a week, eight weeks), overtraining (25 m/min, 60 min/day, 6 days a week, 11 weeks).	Increases IL-10 Increases TNFα, IL-6, interferon-γ, and IL-4.	[53]
Zimmer et al. (2014)	Aerobic	Exercise with 30 min at moderate intensity on a bicycle ergometer.	Increases NK-cells, IL-6, and CD8 (+) T-lymphocytes	[54]

**Table 3:** The importance of nutrition for the immune function.

Name of the Nutrient/Food	Impact on the Immune System	Source
Blueberry	Reduces inflammation and oxidative stress	[78]
Vitamin E	Strengthen the immune system and antioxidative activity	[79]
Papaya	Strengthen the immune system due to its enzymes (Papain), lycopene, carotenoids, alkaloids, monoterpenoids, flavonoids, minerals, and vitamins	[80]
Yogurt	Immune system stimulating effects	[81]
Ginger	Strengthen the immune system	[82]
Green tea	Antioxidant/improves the immune system	[83]
Vitamin D	Regulation of immune system function and proliferation of hematopoietic cells	[84]
Zinc	Improves the immune system and is effective for upper respiratory infections	[85,86]
